# Supplementary material for: Deep neural network trained on gigapixel images improves lymph node metastasis detection in clinical settings
Source: Nat Commun. 2022 Jun 10;13:3347. doi: 10.1038/s41467-022-30746-1 (PMC9187676; doi:10.1038/s41467-022-30746-1)
Supplement: Supplementary file 1 — Supplementary Information [file 41467_2022_30746_MOESM1_ESM.pdf]

Deep neural network trained on gigapixel images improves lymph node metastasis detection in clinical settings

Supplementary Information

Supplementary Methods

Patch-based affine transformation algorithm

The term affine transformation refers to common morphological augmentation steps such as rotation, translation, and scaling. Applying patching in affine transformation is essential for preventing thrashing but is challenging because of the structural changes involved. To overcome this limitation, our proposed method (Fig. 7b) leverages a property of affine transformation where a patch of a transformed image is only associated with an effective region of limited size from the original image. Transforming a small region instead of an entire WSI substantially reduces the memory footprint and thus prevents thrashing. We present detailed procedures for the calculation of the effective region, followed by the remaining steps necessary for obtaining an augmented patch.

Let $\mathbf{I}: \mathbf{R}^2 \rightarrow [0, 1]^3$ denote a WSI, defining the RGB output $\mathbf{I}(\mathbf{v}) \in [0, 1]^3$ given an input coordinate $\mathbf{v} \in \mathbf{R}^2$. Although WSIs as raster graphics store RGB values on grid points, they can be extended to $\mathbf{R}^2$ through interpolation (bilinear interpolation in our implementation) and white-padding. Herein, we define coordinates to be zero centered; that is, the coordinate of the image center is (0, 0). The underlying spatial mapping of coordinates of an affine transformation is defined as $\mathbf{f}(\mathbf{v}) = \mathbf{A}\mathbf{v} + \mathbf{b}$, where an invertible matrix $\mathbf{A} \in \mathbf{R}^{2\times 2}$ and $\mathbf{b} \in \mathbf{R}^2$ are the parameters of $\mathbf{f}(.)$. We denote the transformed image as $\mathbf{I}': \mathbf{R}^2 \rightarrow [0, 1]^3$ such that $\forall \mathbf{v}', \mathbf{I}'(\mathbf{v}') = \mathbf{I}(\mathbf{f}^{-1}(\mathbf{v}'))$.

When a request to access a patch on the transformed image is received, the first step is locating the center of the effective region in the original image. When the transformed patch center is $\mathbf{v}_0'$, the center of the effective region can be obtained by $\mathbf{f}^{-1}(\mathbf{v}_0')$, denoted as $\mathbf{v}_0$. The second step entails calculating the span of the effective region. For the width and height of the requested patch as w' and h', the algorithm calculates both the width and height of the squared effective region by using $a = \sqrt{(w'^2 + h'^2)} / \|\mathbf{A}\|_2$. In the third step, the effective region is cropped out at $\mathbf{v}_0$ with both width and height as a. The cropped effective region represented by $\mathbf{P}: \mathbf{R}^2 \rightarrow [0, 1]^3$ conforms to the equations $\forall \mathbf{v} \in [-a / 2, a / 2]^2, \mathbf{P}(\mathbf{v}) = \mathbf{I}(\mathbf{v} + \mathbf{v}_0)$. The fourth step involves the application of $\mathbf{f}'(\mathbf{v}) = \mathbf{A}\mathbf{v}$, an affine transformation without translation, to the cropped region, thus retrieving a transformed region denoted as $\mathbf{P}': \mathbf{R}^2 \rightarrow [0, 1]^3$, where $\forall \mathbf{v}' \in [-a / 2, a / 2]^2$, $\mathbf{P}'(\mathbf{v}') = \mathbf{P}(\mathbf{f}'^{-1}(\mathbf{v}'))$. Finally, the desired patch with the width w and height h is obtained by centrally cropping the transformed region $\mathbf{P}'(.)$. Although this method for retrieving a transformed patch is more complex than simply cropping the patch from a transformed WSI, their outcomes are equivalent because $\forall \mathbf{v}' \in ([-w / 2, w / 2], [-h / 2, h / 2])$, $\mathbf{P}'(\mathbf{v}') = \mathbf{P}(\mathbf{f}'^{-1}(\mathbf{v}')) = \mathbf{P}(\mathbf{A}^{-1}\mathbf{v}') = \mathbf{I}(\mathbf{A}^{-1}\mathbf{v}' + \mathbf{v}_0) = \mathbf{I}(\mathbf{A}^{-1}\mathbf{v}' + \mathbf{f}^{-1}(\mathbf{v}_0')) = \mathbf{I}(\mathbf{A}^{-1}\mathbf{v}' + \mathbf{A}^{-1}(\mathbf{v}_0' - \mathbf{b})) = \mathbf{I}(\mathbf{A}^{-1}(\mathbf{v}' + \mathbf{v}_0' - \mathbf{b})) = \mathbf{I}(\mathbf{f}^{-1}(\mathbf{v}' + \mathbf{v}_0')) = \mathbf{I}'(\mathbf{v}' + \mathbf{v}_0')$. Furthermore, the present method circumvents the thrashing that is characteristic of affine transformations on WSIs.

Supplementary Tables

Supplementary Table 1 | Extended performance table of our model, the pathologists, and previous models under the main test set, the micrometastasis test subset, and the ITC test subset. The confusion matrices were calculated for our model (at a threshold of 0.4) and pathologists, including the number of true-positive (TP), false-positive (FP), false-negative (FN), and true-negative (TN) LN images. PPV, NPV, and MCC are abbreviations for positive predictive value, negative predictive value, and Matthews correlation coefficient, respectively. Three pathologists (J.L., H.-C.C., and T.-Y.H.) relabeled the 38 equivocal LN images with AI assistance (denoted as partial AI assistance). The data on model performance reported in the bottom two rows of the table were directly retrieved from the publications in question. Considering the between-study discrepancies in test slide distributions, the results may contain bias.

**Model / Pathologist**	**TP**	**FP**	**FN**	**TN**	**Sensitivity**	**Specificity**	**PPV**	**NPV**	**MCC**
The main test set (*n* = 1156)									
Our model	263	12	32	849	0.8915 (0.8503–0.9246)	0.9861 (0.9758–0.9928)	0.9564 (0.9250–0.9773)	0.9637 (0.9491–0.9750)	0.8986 (0.8686–0.9269)
Pathologist S.-C.H.	289	2	6	859	0.9797 (0.9563–0.9925)	0.9977 (0.9916–0.9997)	0.9931 (0.9754–0.9992)	0.9931 (0.9850–0.9975)	0.9818 (0.9681–0.9932)
Pathologist 1	279	2	16	859	0.9458 (0.9134–0.9687)	0.9977 (0.9916–0.9997)	0.9929 (0.9745–0.9991)	0.9817 (0.9705–0.9895)	0.9589 (0.9392–0.9767)
Pathologist 2	286	11	9	850	0.9695 (0.9429–0.9860)	0.9872 (0.9773–0.9936)	0.9630 (0.9347–0.9814)	0.9895 (0.9802–0.9952)	0.9546 (0.9340–0.9731)

Pathologist 3	275	2	20	859	0.9322 (0.8972–0.9581)	0.9977 (0.9916–0.9997)	0.9928 (0.9742–0.9991)	0.9772 (0.9651–0.9860)	0.9497 (0.9284–0.9690)
Pathologist 1 with partial AI assistance	289	2	6	859	0.9797 (0.9563–0.9925)	0.9977 (0.9916–0.9997)	0.9931 (0.9754–0.9992)	0.9931 (0.9850–0.9975)	0.9818 (0.9682–0.9932)
Pathologist 2 with partial AI assistance	291	2	4	859	0.9864 (0.9656–0.9963)	0.9977 (0.9916–0.9997)	0.9932 (0.9756–0.9992)	0.9954 (0.9882–0.9987)	0.9863 (0.9742–0.9956)
Pathologist 3 with partial AI assistance	288	2	7	859	0.9763 (0.9517–0.9904)	0.9977 (0.9916–0.9997)	0.9931 (0.9753–0.9992)	0.9919 (0.9834–0.9967)	0.9795 (0.9648–0.9912)
The micrometastasis test subset (*n* = 919)									
Our model	52	12	6	849	0.8966 (0.7883–0.9611)	0.9861 (0.9758–0.9928)	0.8125 (0.6954–0.8992)	0.9930 (0.9848–0.9974)	0.8432 (0.7668–0.9098)
Pathologist S.-C.H.	57	2	1	859	0.9828 (0.9076–0.9996)	0.9977 (0.9916–0.9997)	0.9661 (0.8829–0.9959)	0.9988 (0.9935–1.0000)	0.9727 (0.9381–1.0000)
Pathologist 1	55	2	3	859	0.9483 (0.8562–0.9892)	0.9977 (0.9916–0.9997)	0.9649 (0.8789–0.9957)	0.9965 (0.9899–0.9993)	0.9537 (0.9086–0.9905)
Pathologist 2	56	11	2	850	0.9655 (0.8809–0.9958)	0.9872 (0.9773–0.9936)	0.8358 (0.7252–0.9151)	0.9977 (0.9915–0.9997)	0.8911 (0.8279–0.9448)
Pathologist 3	52	2	6	859	0.8966 (0.7883–0.9611)	0.9977 (0.9916–0.9997)	0.9630 (0.8725–0.9955)	0.9931 (0.9850–0.9975)	0.9246 (0.8675–0.9713)
Pathologist 1 with partial AI assistance	58	2	0	859	1.0000 (0.9384–1.0000)	0.9977 (0.9916–0.9997)	0.9667 (0.8847–0.9959)	1.0000 (0.9957–1.0000)	0.9820 (0.9532–1.0000)
Pathologist 2	57	2	1	859	0.9828 (0.9076–0.9996)	0.9977 (0.9916–0.9997)	0.9661 (0.8829–0.9959)	0.9988 (0.9935–1.0000)	0.9727 (0.9374–1.0000)

with partial AI assistance									
Pathologist 3 with partial AI assistance	57	2	1	859	0.9828 (0.9076–0.9996)	0.9977 (0.9916–0.9997)	0.9661 (0.8829–0.9959)	0.9988 (0.9935–1.0000)	0.9727 (0.9365–1.0000)
The ITC test subset (*n* = 889)									
Our model	14	12	14	849	0.5000 (0.3065–0.6935)	0.9861 (0.9758–0.9928)	0.5385 (0.3337–0.7341)	0.9838 (0.9729–0.9911)	0.5038 (0.3268–0.6567)
Pathologist S.-C.H.	24	2	4	859	0.8571 (0.6733–0.9597)	0.9977 (0.9916–0.9997)	0.9231 (0.7487–0.9905)	0.9954 (0.9882–0.9987)	0.8861 (0.7836–0.9650)
Pathologist 1	23	2	5	859	0.8214 (0.6311–0.9394)	0.9977 (0.9916–0.9997)	0.9200 (0.7397–0.9902)	0.9942 (0.9865–0.9981)	0.8654 (0.7516–0.9508)
Pathologist 2	27	11	1	850	0.9643 (0.8165–0.9991)	0.9872 (0.9773–0.9936)	0.7105 (0.5410–0.8458)	0.9988 (0.9935–1.0000)	0.8216 (0.7151–0.9102)
Pathologist 3	24	2	4	859	0.8571 (0.6733–0.9597)	0.9977 (0.9916–0.9997)	0.9231 (0.7487–0.9905)	0.9954 (0.9882–0.9987)	0.8861 (0.7844–0.9638)
Pathologist 1 with partial AI assistance	25	2	3	859	0.8929 (0.7177–0.9773)	0.9977 (0.9916–0.9997)	0.9259 (0.7571–0.9909)	0.9965 (0.9899–0.9993)	0.9063 (0.8114–0.9800)
Pathologist 2 with partial AI assistance	26	2	2	859	0.9286 (0.7650–0.9912)	0.9977 (0.9916–0.9997)	0.9286 (0.7650–0.9912)	0.9977 (0.9916–0.9997)	0.9262 (0.8424–0.9850)
Pathologist 3 with partial AI assistance	23	2	5	859	0.8214 (0.6311–0.9394)	0.9977 (0.9916–0.9997)	0.9200 (0.7397–0.9902)	0.9942 (0.9865–0.9981)	0.8654 (0.7551–0.9524)
Related works									
Hu et al.	159	11	21	1025	0.8833	0.9894	0.9353	0.9799	0.8937

					(0.8272–0.9263)	(0.9811–0.9947)	(0.8872–0.9673)	(0.9695–0.9875)	(0.8566–0.9283)
Wang et al.	5217	391	82	9544	0.9845<br>(0.9808–0.9877)	0.9606<br>(0.9566–0.9644)	0.9303<br>(0.9233–0.9368)	0.9915<br>(0.9894–0.9932)	0.9334<br>(0.9275–0.9391)

Supplementary Table 2 | TRIPOD checklist: prediction model development and validation. Items relevant only to the development of a prediction model are denoted by D, items relating solely to a validation of a prediction model are denoted by V, and items relating to both are denoted D;V.

Section/Topic	Item		Checklist Item	Page
**Title and abstract**				
Title	1	D;V	Identify the study as developing and/or validating a multivariable prediction model, the target population, and the outcome to be predicted.	Title page
Abstract	2	D;V	Provide a summary of objectives, study design, setting, participants, sample size, predictors, outcome, statistical analysis, results, and conclusions.	Abstract
**Introduction**				
Background and objectives	3a	D;V	Explain the medical context (including whether diagnostic or prognostic) and rationale for developing or validating the multivariable prediction model, including references to existing models.	Main
	3b	D;V	Specify the objectives, including whether the study describes the development or validation of the model or both.	Main
**Methods**				
Source of data	4a	D;V	Describe the study design or source of data (e.g., randomized trial, cohort, or registry data), separately for the development and validation data sets, if applicable.	Methods: Samples and slide images<br>Methods: Data preparation for model training and evaluation
	4b	D;V	Specify the key study dates, including start of accrual; end of accrual; and, if applicable, end of follow-up.	Methods: Samples and slide images
Participants	5a	D;V	Specify key elements of the study setting (e.g., primary care, secondary care, general population) including number and location of centres.	Methods: Samples and slide images
	5b	D;V	Describe eligibility criteria for participants.	Methods: Samples and slide images
	5c	D;V	Give details of treatments received, if relevant.	Not Applicable
Outcome	6a	D;V	Clearly define the outcome that is predicted by the prediction model, including how and when assessed.	Methods: Overview of the gastric LN assessment workflow<br>Methods: Statistical analysis and evaluation metrics

	6b	D;V	Report any actions to blind assessment of the outcome to be predicted.	Methods: Statistical analysis and evaluation metrics
Predictors	7a	D;V	Clearly define all predictors used in developing or validating the multivariable prediction model, including how and when they were measured.	Methods: Overview of the gastric LN assessment workflow<br>Methods: LN detector<br>Methods: ESCNN for gastric LN metastasis identification<br>Methods: Statistical analysis and evaluation metrics
	7b	D;V	Report any actions to blind assessment of predictors for the outcome and other predictors.	Methods: Statistical analysis and evaluation metrics
Sample size	8	D;V	Explain how the study size was arrived at.	Methods: Data preparation for model training and evaluation
Missing data	9	D;V	Describe how missing data were handled (e.g., complete-case analysis, single imputation, multiple imputation) with details of any imputation method.	Not applicable
Statistical analysis methods	10a	D	Describe how predictors were handled in the analyses.	Methods: Statistical analysis and evaluation metrics
	10b	D	Specify type of model, all model-building procedures (including any predictor selection), and method for internal validation.	Methods: LN detector<br>Methods: ESCNN for gastric LN metastasis identification<br>Methods: Model training
	10c	V	For validation, describe how the predictions were calculated.	Methods: Statistical analysis and evaluation metrics
	10d	D;V	Specify all measures used to assess model performance and, if relevant, to compare multiple models.	Methods: Statistical analysis and evaluation metrics
	10e	V	Describe any model updating (e.g., recalibration) arising from the validation, if done.	Not applicable
Risk groups	11	D;V	Provide details on how risk groups were created, if done.	Not applicable
Development vs. validation	12	V	For validation, identify any differences from the development data in setting, eligibility criteria, outcome, and predictors.	Methods: Data preparation for model training and evaluation
**Results**				

Participants	13a	D;V	Describe the flow of participants through the study, including the number of participants with and without the outcome and, if applicable, a summary of the follow-up time. A diagram may be helpful.	Methods: Data preparation for model training and evaluation
	13b	D;V	Describe the characteristics of the participants (basic demographics, clinical features, available predictors), including the number of participants with missing data for predictors and outcome.	Methods: Data preparation for model training and evaluation
	13c	V	For validation, show a comparison with the development data of the distribution of important variables (demographics, predictors and outcome).	Methods: Data preparation for model training and evaluation
Model development	14a	D	Specify the number of participants and outcome events in each analysis.	Methods: Data preparation for model training and evaluation
	14b	D	If done, report the unadjusted association between each candidate predictor and outcome.	Not applicable
Model specification	15a	D	Present the full prediction model to allow predictions for individuals (i.e., all regression coefficients, and model intercept or baseline survival at a given time point).	Not applicable
	15b	D	Explain how to the use the prediction model.	Results: AI-assisted LN assessment workflow
Model performance	16	D;V	Report performance measures (with CIs) for the prediction model.	Results: ESCNN performance in metastasis identification
Model-updating	17	V	If done, report the results from any model updating (i.e., model specification, model performance).	Not applicable
**Discussion**				
Limitations	18	D;V	Discuss any limitations of the study (such as nonrepresentative sample, few events per predictor, missing data).	Discussion
Interpretation	19a	V	For validation, discuss the results with reference to performance in the development data, and any other validation data.	Results: Comparisons with other weakly supervised methods<br>Results: Impact of image resolutions, data set size, and label types on ESCNN performance
	19b	D;V	Give an overall interpretation of the results, considering objectives, limitations, results from similar studies, and other relevant evidence.	Discussion
Implications	20	D;V	Discuss the potential clinical use of the model and implications for future research.	Discussion
**Other information**				

Supplementary information	21	D;V	Provide information about the availability of supplementary resources, such as study protocol, Web calculator, and data sets.	Supplementary Data 1
Funding	22	D;V	Give the source of funding and the role of the funders for the present study.	Acknowledgments
