# Supplementary material for: Deep neural network trained on gigapixel images improves lymph node metastasis detection in clinical settings
Source: Nat Commun. 2022 Jun 10;13:3347. doi: 10.1038/s41467-022-30746-1 (PMC9187676; doi:10.1038/s41467-022-30746-1)
Supplement: Supplementary file 2 — Reporting Summary [file 41467_2022_30746_MOESM2_ESM.pdf]

nature portfolio

Corresponding author(s): Chao-Yuan Yeh
Last updated by author(s): May 17, 2022

Reporting Summary

Nature Portfolio wishes to improve the reproducibility of the work that we publish. This form provides structure for consistency and transparency in reporting. For further information on Nature Portfolio policies, see our Editorial Policies and the Editorial Policy Checklist.

Statistics

For all statistical analyses, confirm that the following items are present in the figure legend, table legend, main text, or Methods section.

n/a	Confirmed	
☐	☒	The exact sample size (*n*) for each experimental group/condition, given as a discrete number and unit of measurement
☐	☒	A statement on whether measurements were taken from distinct samples or whether the same sample was measured repeatedly
☐	☒	The statistical test(s) used AND whether they are one- or two-sided<br>*Only common tests should be described solely by name; describe more complex techniques in the Methods section.*
☐	☒	A description of all covariates tested
☒	☐	A description of any assumptions or corrections, such as tests of normality and adjustment for multiple comparisons
☐	☒	A full description of the statistical parameters including central tendency (e.g. means) or other basic estimates (e.g. regression coefficient) AND variation (e.g. standard deviation) or associated estimates of uncertainty (e.g. confidence intervals)
☐	☒	For null hypothesis testing, the test statistic (e.g. *F*, *t*, *r*) with confidence intervals, effect sizes, degrees of freedom and *P* value noted<br>*Give P values as exact values whenever suitable.*
☒	☐	For Bayesian analysis, information on the choice of priors and Markov chain Monte Carlo settings
☒	☐	For hierarchical and complex designs, identification of the appropriate level for tests and full reporting of outcomes
☒	☐	Estimates of effect sizes (e.g. Cohen's *d*, Pearson's *r*), indicating how they were calculated

Our web collection on statistics for biologists contains articles on many of the points above.

Software and code

Policy information about availability of computer code

Data collection	Whole-slide images were obtained and digitalized on a NanoZoomer S360 digital slide scanner (Hamamatsu Photonics, Hamamatsu, Japan), and opened using OpenSlide 3.4.1.
Data analysis	The source code of this study can be downloaded from https://github.com/aetherAI/hms2 under the CC BY-NC-SA 4.0 license. The software stack comprised CUDA 11.1 and cuDNN 7.6 for GPU acceleration, PyTorch 1.8.2 and Torchvision 0.9.2 for model construction and training, Open MPI 4.0.1 and Horovod 0.22.1 for multi-GPU training, and OpenCV 4.1.1.26 and Pillow 8.2.0 for image processing. For comparisons among methods, the MIL and MIL-RNN implementation (https://github.com/aetherAI/whole-slide-cnn) and the CLAM implementation (https://github.com/mahmoodlab/CLAM) were used. For statistics, scikit-learn 0.22.1, pROC 1.18.0, epiR 2.0.35, mltools 0.3.5, lme4 1.1.27.1, emmeans 1.6.3, DTComPair 1.0.3, and EnvStats 2.4.0 were used.

For manuscripts utilizing custom algorithms or software that are central to the research but not yet described in published literature, software must be made available to editors and reviewers. We strongly encourage code deposition in a community repository (e.g. GitHub). See the Nature Portfolio guidelines for submitting code & software for further information.

Data

Policy information about availability of data

All manuscripts must include a data availability statement. This statement should provide the following information, where applicable:
- Accession codes, unique identifiers, or web links for publicly available datasets
- A description of any restrictions on data availability
- For clinical datasets or third party data, please ensure that the statement adheres to our policy

The raw experimental data are provided as Source Data, including pathologists' diagnoses and the model-predicted scores on the main test set (Fig. 2 and Table 1),

the throughput and memory consumption of the training methods (Fig. 3), the review time and reported LN counts corresponding to each pathologist-slide obtained from the clinical experiment (Fig. 6). To protect patients' privacy, the slide data are not publicly available. The ImageNet data set used to pre-train models is publicly accessible at https://www.image-net.org .

Field-specific reporting

Please select the one below that is the best fit for your research. If you are not sure, read the appropriate sections before making your selection.

- [x] Life sciences
- [ ] Behavioural & social sciences
- [ ] Ecological, evolutionary & environmental sciences

For a reference copy of the document with all sections, see nature.com/documents/nr-reporting-summary-flat.pdf

Life sciences study design

All studies must disclose on these points even when the disclosure is negative.

Sample size	No sample-size calculation was performed. We retrospectively retrieved slides featuring surgical resections of lymph nodes from gastric carcinoma dissections between 2018 and 2020 from the archive of the Department of Anatomic Pathology at Linkou Chang Gung Memorial Hospital (CGMH) and Kaohsiung CGMH. Sample sizes were chosen to support meaningful conclusions.
Data exclusions	No data were excluded.
Replication	Custom codes are provided for reproducing the results.
Randomization	For the model performance experiment, slide samples in 2019 were randomized fairly into training, validation and test datasets. For the clinical experiment, slide samples from Linkou CGMH in 2020 were shuffled in a random order for S.-C.H. to sample 80 slides. Each pathologist reviewed all 80 slides twice in both the AI-assisted (A) mode and the normal (N) mode. Half of the slides were randomly sampled to be reviewed first in A mode and then in N mode. The remaining 40 slides were reviewed first in N mode and then in A mode. To minimize sampling bias, the order in which the slides were presented differed for each pathologist.
Blinding	For the model performance experiment, no investigator blinding is necessary since our experiments are based on retrospectively collected slide images and software analysis without any human interaction. For the clinical experiment, the dispatching of slide samples for each pathologist and the collection of diagnosis results were performed by software.

Reporting for specific materials, systems and methods

We require information from authors about some types of materials, experimental systems and methods used in many studies. Here, indicate whether each material, system or method listed is relevant to your study. If you are not sure if a list item applies to your research, read the appropriate section before selecting a response.

Materials & experimental systems

n/a	Involved in the study	
[ ]	[x]	Antibodies
[x]	[ ]	Eukaryotic cell lines
[x]	[ ]	Palaeontology and archaeology
[x]	[ ]	Animals and other organisms
[ ]	[x]	Human research participants
[x]	[ ]	Clinical data
[x]	[ ]	Dual use research of concern

Methods

n/a	Involved in the study	
[x]	[ ]	ChIP-seq
[x]	[ ]	Flow cytometry
[x]	[ ]	MRI-based neuroimaging

Antibodies

Antibodies used	Slides were stained for immunohistochemistry (IHC) testing using cytokeratin (clone AE1/AE3, 1:200, Genemed, Torrance, CA, USA), Ep-CAM (clone BerEP4, 1:100, Cell Marque, Rocklin, CA, USA), and calretinin (clone Poly, 1:100, Genemed, Torrance, CA, USA).
Validation	The immunohistochemical procedures were conducted in an automated immunostaining machine (BOND-MAX, Leica Microsystems) with optimal negative and positive controls according to the manufacturers' protocols. Specifically, a slide of control tissue is run in every staining batch. The control tissue is derived from human species and contains both positive and negative staining cells (epithelial cells for cytokeratin, adenocarcinoma for Ep-CAM, and mesothelial cells for calretinin) to serve as both the positive and negative controls.

Human research participants

Policy information about studies involving human research participants

Population characteristics	We retrospectively retrieved slides featuring surgical resections of lymph nodes from gastric carcinoma dissections between 2018 and 2020 from the archive of the Department of Anatomic Pathology at Linkou Chang Gung Memorial Hospital (CGMH) and Kaohsiung CGMH. The detailed characteristics is listed in Fig. 1a.
Recruitment	This study did not involve participant recruitment.
Ethics oversight	The study protocol was approved by the Institutional Review Board (IRB) of CGMH (IRB No. 202000038B0). Written informed consents were waived by the IRB in the study due to the usage of deidentified digital slides.

Note that full information on the approval of the study protocol must also be provided in the manuscript.
