# Supplementary material for: Deep neural network trained on gigapixel images improves lymph node metastasis detection in clinical settings
Source: Nat Commun. 2022 Jun 10;13:3347. doi: 10.1038/s41467-022-30746-1 (PMC9187676; doi:10.1038/s41467-022-30746-1)
Supplement: Supplementary file 3 — Description of Additional Supplementary Files [file 41467_2022_30746_MOESM3_ESM.pdf]

Title: Supplementary Video 1
Description: Brief introduction to the artificial-intelligence-assisted lymph node assessment workflow trained on gigapixel images.
